# Susac Syndrome and Pregnancy

**DOI:** 10.1155/2020/6049126

**Published:** 2020-12-23

**Authors:** Yazan Al-Hasan, Justin L. Hoskin, John C. Wolf, Saha Kamala

**Affiliations:** Barrow Neurological Institute, 350 W Thomas Rd, Phoenix, AZ 85013, USA

## Abstract

Susac syndrome (SuS) is a rare poorly characterised disorder that affects the brain, retina, and cochlea. Here, we present a case of a 31-year-old pregnant female with a new diagnosis of SuS that was successfully managed to 36 weeks of gestation with minimal disease burden to both the mother and newborn. She was treated initially using intravenous methylprednisolone followed by oral prednisone, and intravenous immunoglobulin (IVIg). We stress the importance of a multidisciplinary approach, involving both neurology and maternal-fetal medicine, and provide guidance in navigating the various options for immunosuppressive therapy during pregnancy.

## 1. Introduction

Susac syndrome (SuS) is a rare poorly characterised microangiopathy that causes small infarcts in the brain, cochlea, and retina [1]. Classically, it presents with the clinical triad of visual loss caused by branch retinal artery occlusions (BRAO), sensorineural hearing loss, and subacute encephalopathy [2, 3]. Previous reviews of SuS identified 300–405 cases reported in the literature [4, 5], while a recent estimation of SuS patients in Austria estimates a five-year period prevalence of the disease at 0.148/100,000 and an annual incidence of 0.024/100,000 [6]. Here, we discuss a female pregnant patient with a new diagnosis of SuS.

## 2. Case Presentation

This patient was a 31-year-old right-handed G3P0020 twenty-week pregnant female with a past medical history of idiopathic intracranial hypertension who presented to the emergency department with 4 days of vertigo and worsening vision. At the onset of symptoms, she experienced sudden nonpositional vertigo with severe nausea as well as decreased hearing in the left ear. She also noted nystagmus when looking in the mirror. Her symptoms improved mildly over the course of the day, so she did not seek immediate medical advice.

Two days following the initial onset, her symptoms returned, at which point she was evaluated at an outside emergency department. She was diagnosed with vertigo and discharged with prescriptions for meclizine and metoclopramide. Her symptoms then progressed to involve peripheral vision loss bilaterally, for which she was examined by an ophthalmologist. On dilated ophthalmic exam, she was found to have multiple areas of retinal ischemia and she was referred to our emergency department for further evaluation.

**Figure 1:**
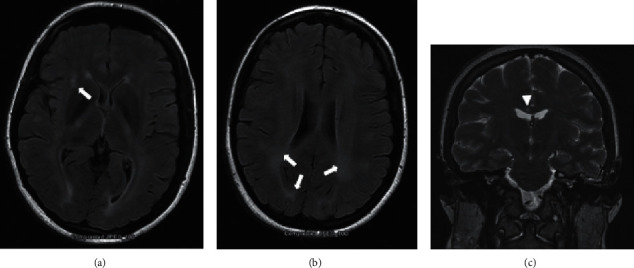
(a) and (b) Axial MRI brain without contrast demonstrating patchy and confluent FLAIR hyperintensity throughout the white matter, markedly abnormal for age (arrows). (c) Coronal MRI brain FLAIR sequence demonstrating a lesion in the corpus callosum (arrowhead), performed prior to her presentation to our facility.

**Figure 2:**
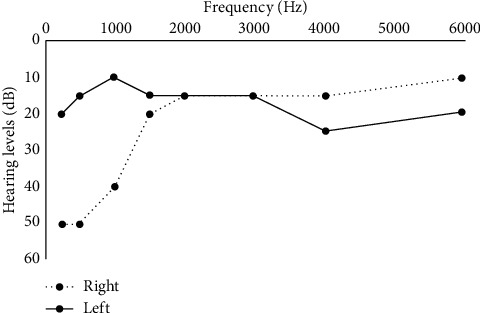
Audiogram results demonstrating low-frequency sensorineural hearing loss in the right ear (dotted line) with her left ear (solid line) unaffected.

On magnetic resonance imaging (MRI) brain without contrast, nonspecific diffuse white matter signal abnormality was observed (Figures 1(a) and 1(b)), which was considered markedly abnormal for the patient's age. A bedside lumbar puncture was performed, and cerebral spinal fluid analysis showed glucose 58 mg/dL, protein 48 mg/dL, nucleated count 2 per uL, oligoclonal bands negative, and myelin basic protein 8.92 ng/mL. In addition, the patient had an MRI brain with and without contrast performed a year earlier that mentioned white matter lesions in the corpus callosum (Figure 1(c)). She also had with her an audiogram from a year earlier showing low frequency sensorineural hearing loss in the right ear (Figure 2). Given the constellation of symptoms (vertigo, hearing loss, and visual disturbances) in conjunction with her MRI changes, eye exam findings, and audiometric testing results, a diagnosis of SuS was established.

She was treated with methylprednisolone 1 gm IV for 3 days followed by oral prednisone 50 mg daily. Additionally, she was started on intravenous immunoglobulin (IVIg) 0.5 gm/kg for 4 days for a total of 2 gm/kg. During admission, she noted improvement, with near resolution of her vertiginous symptoms as well as some improvement in her hearing. In conjunction with her maternal-fetal medicine physician and her obstetrician, the plan at discharge was to continue prednisone 50 mg daily and maintenance IVIg 0.5 gm/kg for 2 days every 3 weeks. The decision to start steroid sparing immunosuppressants was delayed per patient preference given her pregnancy status.

After discharge, she returned to the hospital three weeks later for one additional round of IVIg and continued on oral prednisone for the duration of her pregnancy. A cesarean section was performed at 36 weeks without complications of SuS to the mother and child. She continued to follow up with neurology for serial imaging and initiation of steroid sparing immunotherapy, namely, rituximab.

## 3. Discussion

### 3.1. Pathophysiology

SuS is a rare condition whose definitive etiology remains unknown [3, 7]. At this time, it is classified as an immune-mediated, occlusive microvascular endotheliopathy and/or basement membranopathy that results in endothelium-induced microvascular occlusion in the central nerve system, inner ear, and retina [2, 4]. It has also been termed small infarctions of cochlear, retinal and encephalic tissue (SICRET syndrome), and retinocochleocerebral vasculopathy [8, 9]. As mentioned in the introduction to this paper, SuS is a rare condition and the prevalence is likely underreported. Women seem to be more commonly affected than men at a rate of 3 : 1, and most cases involve Caucasian individuals, though this may be related to medical discrepancies [4]. The estimated mean age of onset is approximately 31.6 years, ranging from 8 to 65 [2, 4, 10–12].

Previous theories regarding pathophysiology suggested that the syndrome is an autoimmune vasculitis that affects the endothelium likely mediated by antiendothelial cell antibodies (AECAs) [13, 14]. AECAs were thought to cause activation of the complement system leading to subsequent deposition of C4d, basement membrane thickening, and endothelial necrosis [10]. Evidence supporting this etiology included SuS patients with high serum AECA, elevated factor VIII (released by damaged endothelium), and tissue pathology with endothelial cell necrosis, basement membrane thickening, and C3d and C4d deposition in vessel walls [10]. However, a recent study showed that only 30% of patients with a definite SuS had elevated serum AECA, suggesting that AECA may represent an epiphenomenon [15]. Interestingly, data presented by Gross et al. strongly implicate cytotoxic T-lymphocyte adhesion to CNS microvessels leading to endotheliopathy in both pathological specimens and a mouse model of SuS [16]. Furthermore, they also report the use of natalizumab, an inhibitor of T-cell adhesion, ameliorated symptoms in four SuS patients.

### 3.2. Disease Course

The syndrome typically presents as a clinical triad of encephalopathy, sensorineural hearing loss, and visual disturbances secondary to BRAO [1, 3]. There is a large variability on initial presentation; some estimates suggest that at the time of initial evaluation, less than 20% of patients exhibit the full clinical triad, while other reports suggest that 81% of cases have the complete triad at onset [2–4]. However, estimates suggest that during the clinical course, 70–98% of patients will develop the full triad of symptoms [3, 4]. At presentation, CNS involvement was the most frequent manifestation (80%), followed by ocular involvement (50%) and finally auditory involvement (30%) [3, 4, 9].

Classically, encephalopathy was described as cognitive impairment or psychosis, often leading to patients undergoing psychiatric evaluation [1]. However, more recent articles have discussed encephalopathy as “brain involvement,” widening the symptom inclusion criteria to headache, motor and sensory deficiencies, aphasia, cognitive impairment, emotional disturbances, apathy, psychosis, urinary insufficiency, and others [4, 17].

For the criterion of vestibulocochlear involvement, the patient should have at least one of the following symptoms: hearing loss, tinnitus, or vertigo [17–19]. By far, hearing loss is the most common vestibulocochlear disorder identified; one review identified hearing loss in 93% of patients [19]. The hearing loss can be either unilateral or bilateral and is often described as low-frequency sensorineural hearing loss [2, 19, 20].

The manifestations of BRAO vary greatly from subtle to expansive, unilateral, or bilateral [3]. The most common complaints are segmental loss of vision in one or both eyes as well as visual scintillating scotomas [21]. More recently, Gass plaques (retinal arterial wall plaques) have been described in fluorescein staining of retinal arterioles which can help confirm the diagnosis of SuS [18]. In addition to the classic triad of symptoms, a plethora of other symptoms have been found to occur frequently in these patients including headache, myalgia, and dermatological signs [4, 22]. Headache is often present on disease onset and resembles migraine headache [17]. While SuS is a rare condition, it is an important differential diagnosis for several neurologic, psychiatric, otolaryngologic, and ophthalmologic conditions [23].

Similar to the variability in the initial presentation, the disease course can be volatile as well. At one end of the spectrum, patients may present with mild and brief (less than a year) disease. These patients may experience full reversible ischemia in the brain, retina, and cochlea [2]. Occasionally, these patients acquire little to no residual disability. In contrast, patients with severe SuS experience a prolonged disease course that lasts for years and can lead to permanent ischemic damage [2]. The primary organs impacted by SuS, the brain, retina, and cochlea, are highly susceptible to ischemic damage. In addition to the variability in severity, the disease can vary in its remission status. Some suggest that SuS can be monocyclic, polycyclic, and chronic-continuous, with a time frame of 2 years being used to separate the monocyclic course from the other forms [4, 21, 24]. It is likely that there is no pathological difference between these forms of SuS and that this represents a relapsing-remitting course [5]. Separating them into different entities may be more deleterious for patients and confusing for providers. Of note, there are reports of recurrence of SuS after years of remission [25, 26].

### 3.3. Diagnosis

There are several diagnostic modalities that help confirm the diagnosis SuS. Arguably the most important diagnostic procedures are MRI of the brain, audiometric testing, and retinal fluorescein angiography [27]. Classically, the corpus callosum has been found to have T2 hyperintensities that resemble “snow balls” [28]. These lesions are thought to occur as a result of infarction of tiny arterioles in the central part of the corpus callosum. T2-weighted images may demonstrate small multifocal hyperintensities in the periventricular regions and centrum semiovale. These SuS lesions tend to be more punctate than the large ovoid lesions often identified with multiple sclerosis (MS) lesions [28]. Audiogram testing can show bilateral sensorineural hearing loss predominantly in the low frequencies [2]. Funduscopy and fluorescein retinal angiography may demonstrate unilateral or bilateral distal BRAO [12]. Some estimates suggest that BRAOs are detected in 99% of patients [4].

Laboratory studies including specific serological markers for SuS do not currently exist. As discussed, AECA titers have been shown to be elevated in only 26% of patients. Moderate elevation of protein was the most common CSF finding in SuS, observed in 84% of cases with available CSF data [4, 15, 29]. While the presence of oligoclonal bands or elevation in IgG in the CSF does not exclude SuS, their absence can help differentiate this disease from MS [4, 20]. Additional diagnostic tests including comprehensive screening for autoantibodies, blood clotting abnormalities, CSF analysis, EEG, cerebral catheter angiography, and leptomeningeal biopsy have not proven to provide clinically relevant information [4]. Interestingly, our patient had both vestibular as well as cochlear involvement and therefore not only was an audiogram performed but videonystagmography (VNG) as well. This demonstrated a significant unilateral vestibulopathy that appeared partially compensated, reflecting her recent onset of improving vertigo. We recommend performing a VNG in all patients with vestibular symptoms and also recommend using this test to monitor patients over time who may present with vertigo upon relapse.

### 3.4. Treatment

Due to the rarity of SuS, the great variability of disease presentation and severity, and the lack of adequate objective biomarkers of disease activity, the development of a treatment guideline with a randomized control trial has been challenging [2]. Based on the current understanding of pathogenesis, the primary target in treatment is the immune system, often with immunosuppression [12, 24]. The most recent guidelines are dependent on the symptoms at presentation. For example, a patient with severe encephalopathy may require more aggressive and prolonged treatment than a patient who has mild-moderate hearing loss or BRAO [2].

While treatment must be tailored to the individual patient, the mainstays have included corticosteroids, IVIg, and rituximab, plus one or two additional immunomodulating drugs (methotrexate, mycophenolate, tacrolimus, infliximab, or cyclophosphamide) [30]. Corticosteroids can be given intravenously in the emergent setting with methylprednisolone 1 g/day before switching to an oral prednisone taper based on symptom severity [2, 18]. IVIg is commonly started at 2 g/kg given over several days, with maintenance IVIg then given every 2–4 weeks, for a total of 6 months. From there, the most common medication choices tend to be rituximab and mycophenolate, with one report suggesting initiation of these medications at the onset of symptoms and another recommending reserving these medications for breakthrough symptoms only [2, 18]. The ability to distinguish between a diagnosis of MS and SuS is important as some MS treatments may worsen SuS [31]. Eventually, these medications may be tapered off if symptoms improve or stabilize and the MRI remains inactive. Of note, patients with primary retinal disease may have difficulty tapering off IVIg as this medication has been found to be quite effective at preventing retinal relapses [18].

### 3.5. Susac and Pregnancy

Reported cases of SuS during pregnancy are rare with an estimate of 5% of cases occurring in association with pregnancy [4, 13, 22, 26, 27, 32–39]. Of the reported cases, the behavior of SuS during pregnancy has been heterogeneous and no statistically significant characteristics have been established [13]. The pathophysiology of disease onset remains unclear; however, it is reasonable to propose that hormonal and immunological changes associated with pregnancy as well as a transient hypercoagulable state may unmask the disease or induce a flare [3, 30, 31]. Recurrence of SuS is also reported in patients undergoing hormone replacement therapy [25], further emphasizing the hormonal contribution to SuS pathophysiology. This complex relationship between autoimmune disease and pregnancy is not unusual and is also seen in other autoimmune diseases including rheumatoid arthritis, thyroid disease, and others [40, 41]. In the postpartum period, there are reports of worsening encephalopathy as well as a reported case of SuS exacerbation occurring after an aborted pregnancy [30]. Despite the increased risk of SuS associated with pregnancy, it is possible for patients in remission to have a safe pregnancy without flare [42]. The pathophysiology behind this relationship is unknown, but we hypothesize that there may be a hormonal influence on the patient's immunity.

As discussed, treatment guidelines for SuS are suggested, but these guidelines have potential limitations with pregnant patients. Careful discussion with the patient and maternal-fetal medicine specialists is warranted. A list of possible treatment options and their risk in pregnancy are discussed below (Table 1) [37, 45–50]. Our patient was started on IVIg in the acute setting and received one maintenance IVIg dose [2]. She showed nearly immediate improvement with her first doses of IVIg. She was also maintained on prednisone 50 mg daily for a long duration (14 weeks) to aid with symptom control. While there is no treatment guideline for SuS in pregnancy, other cases have demonstrated a positive response to IVIG as well [13]. The overall prognosis of SuS and pregnancy remains unknown.

## 4. Conclusion

SuS is a rare neurologic disease with a variable course. Treatment regimens have been suggested, but it is unclear what treatment options are best for pregnant patients with SuS. For our patient, she had an initial positive response to IVIg and required a maintenance dose 3 weeks after her initial dose. There was a discussion with the patient regarding addition of rituximab to her regimen, but she decided to forego that treatment until after delivery. She underwent an elective cesarean section at week 36 of pregnancy without complications. As discussed, there may be an exquisite response to IVIg in SuS. We recommend that patients be started quickly on IVIg and that it be continued throughout their pregnancy with maintenance dosing every 2–4 weeks based on the patient's clinical course. Intravenous methylprednisolone with maintenance oral prednisone is also relatively safe and is a rapid bridge to IVIg therapy. Additional steroid sparing immunosuppressants such as rituximab can be considered, though this discussion should involve a multidisciplinary approach.

## Figures and Tables

**Table 1:** Summary of several medications that are used in the treatment of Susac syndrome and their risk categories established by the United States Food and Drug Administration.

Agent	Pregnancy category	Comments
Corticosteroids	C/D C	Maternal and fetal risks include hyperglycemia, hypertension, and adrenal axis suppression Fetal risk of cleft lip and low birth weight
Prednisone		
Methylprednisolone		
IVIg	C	Limited data. Crosses the placenta, and it is used to treat some neonatal conditions
Infliximab	Low-to-moderate risk [43]	Crosses the placenta. Report of agranulocytosis in infants [44]
Cyclophosphamide	D	If exposed in the first trimester, reports of skeletal malformations, leukopenia, anemia, pancytopenia, severe bone marrow hypoplasia, and gastroenteritis
Azathioprine	D	Reports of congenital anomalies, immunosuppression, lymphopenia, pancytopenia, and intrauterine growth retardation
Mycophenolate mofetil	D	Associated with congenital malformations and first-trimester pregnancy loss
Rituximab	C	Limited data available; transient B-cell lymphocytopenia
Plasmapheresis	Unknown	Potential maternal risk during plasmapheresis does not seem to increase as a result of pregnancy
Aspirin	D	Likely safer in low doses Increased perinatal mortality, delayed closure of ductus arteriosus, and intrauterine growth retardation
Gadolinium (IV)	C	Crosses the placenta. May be an increased risk for rheumatological, inflammatory, or infiltrative skin conditions
Cyclosporine	C	May result in low birth weight and premature births
Methotrexate	X	Can cause teratogenic effects, embryotoxicity, abortion, and fetal defects. Contraindicated during pregnancy

A = controlled studies show no fetal risk. B = animal studies do not demonstrate risk. There is no evidence of risk in humans. C = animal studies show adverse reaction on the fetus, no studies in humans. D = positive evidence of human fetal risk. X = contraindicated in pregnancy.

## Data Availability

No data were used to support the findings of this study.

## Abbreviations

SuS: Susac syndrome
BRAO: Branch retinal artery occlusion
AECA: Antiendothelial cell antibody
IVIG: Intravenous immunoglobulin
MS: Multiple sclerosis
MRI: Magnetic resonance imaging
VNG: Videonystagmography.
